# Pancreatic Pseudocyst Filled with Biliary Stones Mimicking a Pancreatic Groove Tumor: A Case Report

**DOI:** 10.70352/scrj.cr.25-0202

**Published:** 2025-09-09

**Authors:** Hiromitsu Imataki, Masaoki Hattori, Akihiro Hirata, Akihiro Tomida, Jumpei Shibata, Marika Suzuki, Hideharu Shintomi, Motoi Yoshihara, Tomoaki Takeyama, Yoshikazu Mizoguchi

**Affiliations:** 1 Department of Surgery, Nishichita General Hospital, Tokai, Aichi, Japan; 2 Department of Gastroenterology, Nishichita General Hospital, Tokai, Aichi, Japan; 3 Department of Pathology, Nishichita General Hospital, Tokai, Aichi, Japan

**Keywords:** common bile duct, fistula, hemorrhage, pancreatic pseudocyst, stone, groove pancreatitis

## Abstract

**INTRODUCTION:**

Reports of fistulas in the common bile duct caused by pancreatic pseudocysts have increased. However, to the best of our knowledge, no prior report has described pseudocysts filled with microbiliary stones that were difficult to differentiate from neoplastic lesions.

**CASE PRESENTATION:**

A 64-year-old man presented with groove pancreatitis attributable to heavy alcohol consumption and a hypovascular mass in the groove area with duodenal bleeding. The lesion, which was initially considered a pancreatic groove tumor with groove pancreatitis, was treated with subtotal stomach-preserving pancreaticoduodenectomy. However, a post-surgical pathological analysis revealed that it was a pancreatic pseudocyst in the groove area containing bilirubin calcium stones and pancreatic stones.

**CONCLUSIONS:**

Cases comprising fistula formation in the common bile duct are rare. A pancreatic pseudocyst that formed a fistula with the common bile duct was suspected in the present case. This case was unique because the pseudocyst was filled with microbiliary stones. This report highlights the difficulty in differentiating a pseudocyst filled with bilirubin calcium stones from a neoplastic lesion and underscores the importance of the accurate diagnosis and management of this rare pathology.

## Abbreviations

ERCP
: endoscopic retrograde cholangiopancreatography
EUS
: endoscopic ultrasound
GI
: gastrointestinal
IPMC
: intraductal papillary mucinous carcinoma
IPMN
: intraductal papillary mucinous neoplasm
MRCP
: magnetic resonance cholangiopancreatography
SSPPD
: subtotal stomach-preserving pancreaticoduodenectomy

## INTRODUCTION

Pancreatic pseudocysts develop as a result of inflammation or trauma and consist of damaged or necrotic tissue, blood, and exudates. Such pseudocysts are encapsulated by granulation tissue comprising fibrous connective tissue that forms as a result of the inflammatory response. Fistula formation involving adjacent organs is a known complication of pancreatic pseudocysts.^1,2)^ Although rare, reports of fistulas involving the common bile duct are increasing.^3)^

Groove pancreatitis is a rare form of chronic focal pancreatitis that affects the anatomical groove between the pancreatic head, duodenum, and common bile duct and is classified into pure and segmental types.^4,5)^ Embryologically, the lesion is located in the region corresponding to the accessory pancreatic duct. Key features include chronic inflammation of the duodenal wall with fibrosis and stenosis, Brunner’s gland hyperplasia, and the presence of cystic lesions such as true cysts or pseudocysts.^6)^ Additionally, tapering and varying degrees of stenosis of the common bile duct are commonly observed.^7)^

We present the case of a hypovascular mass in the groove area of a patient with groove pancreatitis caused by heavy alcohol consumption. The mass was initially diagnosed as a pancreatic groove tumor with groove pancreatitis; therefore, subtotal stomach-preserving pancreaticoduodenectomy (SSPPD) was performed. A postoperative pathological examination revealed that the lesion was a pseudocyst filled with fine biliary stones and pancreatic stones in the groove area. Although reports of pancreatic pseudocysts that perforate the common bile duct have been increasing, to the best of our knowledge, no prior report has described pseudocysts filled with microbiliary stones that were difficult to differentiate from neoplastic lesions.

## CASE PRESENTATION

A 64-year-old man was referred to our hospital after a routine health screening yielded positive fecal occult blood test results. His medical history included hyperlipidemia, hyperuricemia, cervical spondylosis, and surgery for a chronic subdural hematoma. The patient consumed approximately 900 mL of sake daily (ethanol equivalent, 110 g/day).

**Fig. 1:**
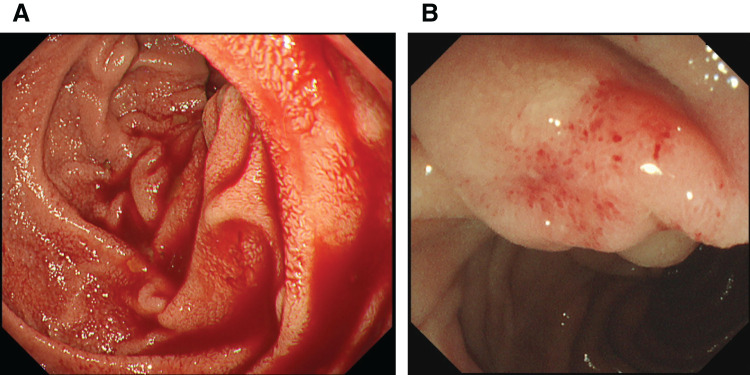
Upper gastrointestinal endoscopy images. (**A**) Active bleeding is observed in the descending part of the duodenum at the time of hospital presentation. (**B**) Edematous changes appear in the mucosa surrounding the minor duodenal papilla.

Upper gastrointestinal (GI) endoscopy was performed because of suspected upper GI bleeding and revealed active bleeding with an unclear origin in the descending duodenum and edematous changes in the mucosa around the minor papilla. Abnormalities such as bleeding were not observed in the major papilla (**Fig. 1**).

**Fig. 2:**
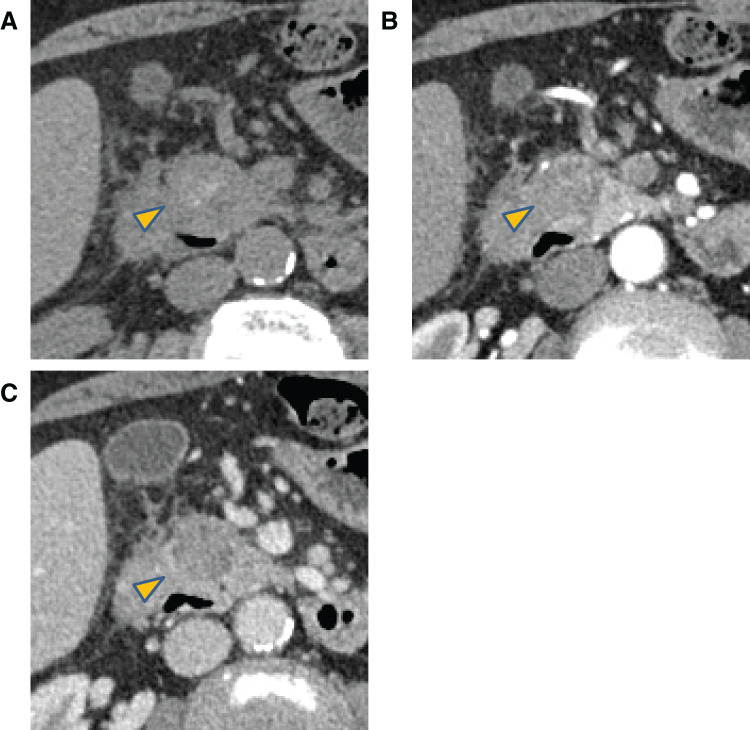
Dynamic CT images. (**A**) Image obtained during CT without contrast. (**B**) Arterial phase image. (**C**) Portal phase image. A 24-mm × 19-mm mass (arrowhead) with high attenuation areas and poor internal contrast enhancement is observed in the groove area. Contrast enhancement is observed during the delayed phase in the peripheral region of the mass. Additionally, increased fat density is observed around the descending part of the duodenum adjacent to the mass; however, no evidence of contrast agent extravasation is observed.

Plain CT revealed high attenuation in the groove area. Dynamic CT showed a 24- × 19-mm hypovascular mass with areas of high attenuation in the groove area and late-phase peripheral enhancement. Increased adipose tissue density around the descending duodenum adjacent to the mass was also observed; however, no evidence of contrast extravasation was observed (**Fig. 2**).

**Fig. 3:**
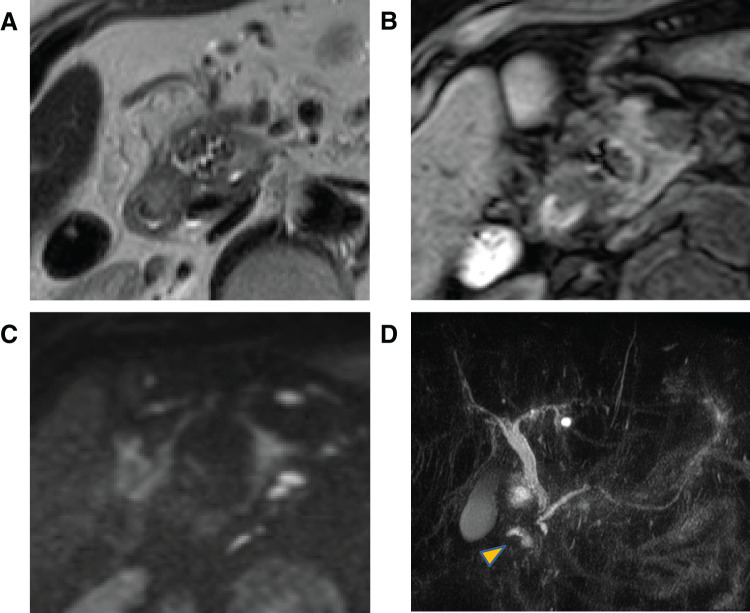
MRI images. (**A**) Image obtained using T2-weighted MRI. (**B**) Image obtained using T1-weighted MRI. (**C**) Image obtained using DWI. (**D**) MRCP image (arrowhead: mass). The groove area mass exhibits internal characteristics of high signal, low signal, and high signal intensity during T2-weighted, T1-weighted, and DWI imaging, respectively. Additionally, small clustered nodules exhibiting low signal intensity during T2-weighted MRI, T1-weighted MRI, and DWI are observed. MRCP reveals no dilation or irregularity in the main pancreatic duct. Pancreaticobiliary maljunction is not observed. Gallbladder and common bile duct stones are absent. Tapering of intrapancreatic bile duct is observed. DWI, diffusion-weighed imaging; MRCP, magnetic resonance cholangiopancreatography

Both T1-weighted and T2-weighted MRI and diffusion-weighted imaging revealed that the groove area mass contained clustered hypointense nodules. Additionally, T2-weighted MRI and diffusion-weighted imaging indicated that the mass exhibited hyperintense content, whereas T1-weighted MRI indicated hypointense content. Magnetic resonance cholangiopancreatography (MRCP) revealed no dilation or irregularity in the main pancreatic duct, no evidence of pancreaticobiliary maljunction, and no stones in the gallbladder and common bile duct; however, tapering of the intrapancreatic bile duct was revealed (**Fig. 3**).

**Fig. 4:**
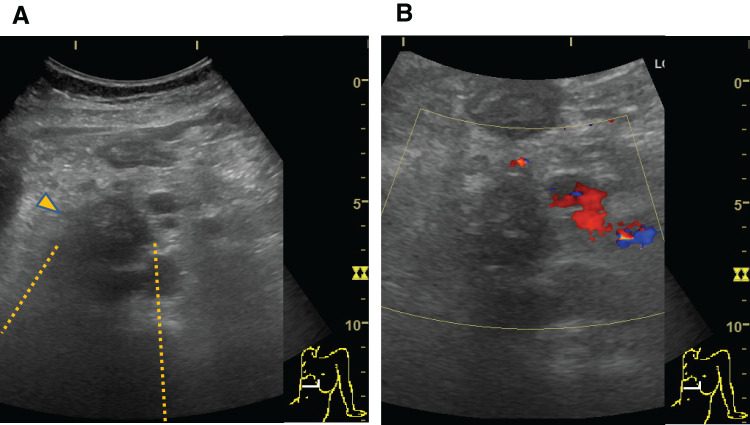
Abdominal ultrasonography images. (**A**) Abdominal ultrasonography image revealing a heterogeneous hypoechoic mass in the groove area. High-echo structures within the mass are accompanied by acoustic shadowing. Arrowhead: mass. Dotted line: acoustic shadowing. (**B**) Color Doppler examination image showing no Doppler signals within the mass.

Abdominal ultrasonography revealed a hypoechoic heterogeneous mass in the groove area that contained high-echo structures with acoustic shadows. Doppler signals were not detected within the mass (**Fig. 4**).

**Fig. 5:**
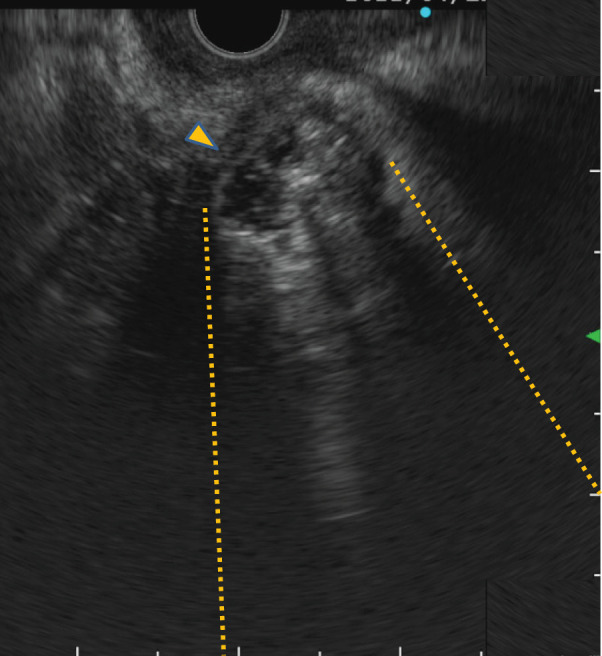
Endoscopic ultrasonography image. The mass has well-defined margins, internal heterogeneity, and a hypoechoic rim at the periphery. Internally, the mass shows areas of high echogenicity with accompanying acoustic shadowing as well as regions of anechoic areas. Arrowhead: mass. Dotted line: acoustic shadowing.

Endoscopic ultrasound (EUS) revealed a well-defined, heterogeneous, hyperechoic mass with some anechoic areas that suggested a solid lesion with cystic components. Therefore, EUS-guided tissue acquisition was performed transduodenally (**Fig. 5**). Cytology indicated epithelial proliferation suggestive of an intraductal papillary mucinous neoplasm (IPMN) and brown amorphous material.

Blood tests revealed normal amylase and lipase levels and the absence of significant abnormalities. Tumor markers, including carcinoembryonic antigen and carbohydrate antigen 19-9, were also within normal limits (**Table 1**).

**Table 1:** Laboratory data at the time of admission

WBC	4300/mm^3^	LDH	172 IU/L
RBC	515 × 10^4^/mm^3^	BUN	17 mg/dL
Hb	13.1 g/dL	Cre	0.85 mg/dL
PLT	35.2 × 10^4^/μL	eGFR	70.2 mL/min/1.73 m^2^
CRP	0.1 mg/dL	AMY	43 U/L
TP	6.8 g/dL	LIP	47 U/L
Alb	4.2 g/dL	Glu	152 mg/dL
T-Bil	0.4 mg/dL	HbA1c	5.9%
AST	26 U/L	CEA	2.8 ng/mL
ALT	19 U/L	CA19-9	4.3 U/mL

Blood test results revealed no significant abnormalities. Amylase and lipase levels were within the normal ranges. Levels of tumor markers such such as CEA and CA19-9 were not elevated.

Alb, albumin; ALT, alanine transaminase; AMY, amylase; AST, aspartate transaminase; BUN, blood urea nitrogen; CA19-9, carbohydrate antigen 19-9; CEA, carcinoembryonic antigen; Cre, creatinine; CRP, C-reactive protein; eGFR, estimated glomerular filtration rate; Glu, glucose; Hb, hemoglobin; HbA1c, hemoglobin A1c; LDH, lactate dehydrogenase; LIP, lipoprotein; PLT, platelet; RBC, red blood cell; T-Bil, total bilirubin; TP, total protein; WBC, white blood cell

Based on these findings, a pancreatic groove tumor and groove pancreatitis were suspected. Epithelial proliferation suggestive of IPMN was observed via EUS-guided tissue acquisition, thus leading to the suspicion of an atypical IPMN or an atypical intraductal papillary mucinous carcinoma (IPMC) as the most likely diagnosis. Because of the possibility of an IPMN and the need for hemostasis, we decided to proceed with SSPPD. Additionally, because of the possibility of an IPMC, lymph node dissection was performed. Furthermore, because the benefit of neoadjuvant chemotherapy for confirmed IPMC remains unclear, we chose not to administer preoperative chemotherapy. Because we chose this strategy, we decided not to repeat tissue sampling. Obstructive jaundice and common bile duct dilation were not observed, and CT and MRI showed no evidence of communication with the main pancreatic duct; therefore, endoscopic retrograde cholangiopancreatography (ERCP) and pancreatic juice cytology were not performed. Although a definitive preoperative diagnosis was not established, surgery was performed 42 days after the initial presentation because of potential malignancy and the risk of recurrent GI bleeding. Oral iron therapy was initiated on the day of the diagnosis of duodenal bleeding; therefore, it was not possible to assess rebleeding based on stool characteristics. Despite iron supplementation, the patient’s hemoglobin level temporarily decreased from 11.0 g/dL at presentation to 10.3 g/dL.

During SSPPD, intraoperative findings included a firm pancreas attributable to chronic inflammation and adhesions surrounding the vascular and other tissues. The mass in the groove area was firmer than the pancreatic parenchyma. Reconstruction was performed using a modified Child method. The operative time was 13 h and 20 min, and the estimated blood loss was 4442 mL. The surgical duration was prolonged because of bleeding from a superior mesenteric artery branch that occurred as a result of severe adhesions caused by chronic pancreatitis. A pancreatic fistula developed postoperatively; however, it resolved with conservative treatment. The patient was discharged on POD 44. At 2.5 years postoperatively, the patient was free of any abnormalities.

**Fig. 6:**
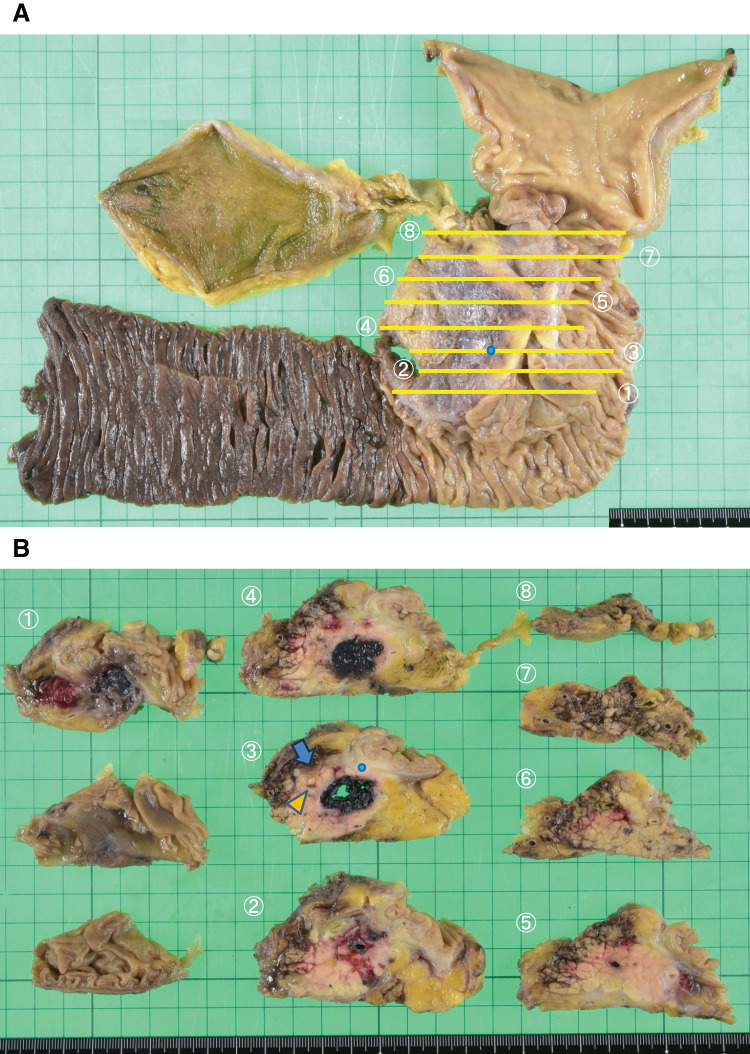
Gross specimen and IPMN mapping. (**A**) Unsliced specimen. (**B**) Sliced specimen. A cystic structure is observed within the groove area, and the cyst cavity is filled with small black stones. The IPMN is located separately from the cystic structure. Arrow: common bile duct. Arrowhead: main pancreatic duct. Blue dot: IPMN. IPMN, intraductal papillary mucinous neoplasm

**Fig. 7:**
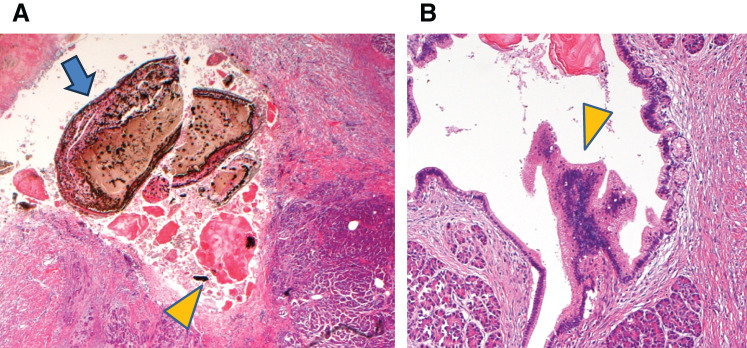
Histopathological findings related to stones and a neoplasm. (**A**) The black calculi are bilirubin calcium gallstones (arrow). Some eosinophilic pancreatic stones are also observed (arrowhead). (**B**) An IPMN appears in the branches of the pancreatic duct (arrowhead). IPMN, intraductal papillary mucinous neoplasm

A macroscopic examination of the resected specimen revealed a cystic structure filled with a small amount of clear fluid and numerous black pebble-like stones in the groove area (**Fig. 6**). A pathological examination confirmed that the cyst lacked an epithelial lining, thus leading to the diagnosis of a pancreatic pseudocyst. The black stones (diameter, 1–3 mm) were identified as bilirubin calcium stones (95%) and eosinophilic pancreatic stones (5%). Bilirubin calcium stones were characterized by the formation of 2 to 3 black outer rings and positive Masson-Fontana staining, melanin bleaching, and bile staining results (**Fig. 7**). A histopathological examination of the pancreatic parenchyma in the groove area revealed features of chronic pancreatitis with fibrosis and marked active inflammation, particularly around the pancreatic pseudocyst. The duodenal wall exhibited active inflammation, hemorrhage, fibrosis, thickening, and Brunner’s gland hyperplasia, which were consistent with groove pancreatitis. Additionally, inflammatory changes were observed in the pancreatic head outside the groove area, thus leading to the classification of the segmental form of groove pancreatitis. An IPMN detected in a pancreatic duct branch (**Fig. 7**) was classified as hyperplastic, and immunohistochemical staining indicated the following results: CDX2^–^, MUC1^−^, MUC2^−^, MUC5AC^−^, and MUC6^+^. Based on these findings, the lesion did not conform to any of the established IPMN subtypes. Additionally, a fistula involving the common bile duct was not identified, and pancreaticobiliary maljunction was ruled out after examination of the resected specimen. A re-evaluation of the tissue acquired using EUS suggested that the amorphous brown material comprised fragments of bilirubin calcium stones.

## DISCUSSION

Pancreatic enzyme leakage caused by inflammation-mediated or trauma-mediated disruption of the pancreatic duct typically results in the formation of pancreatic pseudocysts. Pancreatic pseudocysts are frequently encountered in the groove area and are associated with groove pancreatitis.^6)^ Complications of pancreatic pseudocysts include hemorrhage, infection, obstructive jaundice, and portal vein obstruction.^1,2)^ Fistula formation involving the stomach, colon, duodenum, and spleen has been well-documented. Cases involving the common bile duct are rare; however, a PubMed search of articles published between 1999 and 2024 that included the keywords “pancreatic pseudocyst,” “common bile duct,” “fistula,” “biliopancreatic fistula,” “pancreaticobiliary fistula,” and “gallstone” identified 14 cases (**Table 2**) of pancreatic pseudocysts that perforated the common bile duct or formed a fistula.^3,8–18)^

**Table 2:** Cases of pancreatic pseudocysts with a fistula involving the common bile duct (1999–2024)

Year	Author	Age (years)	Sex	Etiology	Hemorrhage	Fistula detected by	Location of stones	Treatment
2000	Miyanishi^8)^	65	Male	Alcoholic	—	ERCP	CBD stones	F/U, spontaneousremission
2001	Boulanger^9)^	52	Male	Alcoholic	—	ERCP	—	EBS
2001	Carrere^10)^	74	Male	Alcoholic	—	ERCP	—	EPST and EBS
	Carrere^10)^	67	Male	Acute	Intracystic	ERCP	CBD stones	PP jejunostomy, hepaticojejunostomy, and cholecystectomy
	Carrere^10)^	65	Male	Acute	—	ERCP	—	Cholecystectomy and biliary and pancreatic bypass
2004	Apel^11)^	51	Male	Alcoholic	—	ERCP	—	EPS
2006	Ragunath^12)^	39	Male	Acute/pancreaticobiliary maljunction	—	ERCP	—	EPS
2006	Rickes^3)^	67	Male	Alcoholic	—	ERCP	—	EBS
2008	Miyatani^13)^	57	Male	Acute	—	ERCP	—	EPS and choledochojejunostomy
2009	Al Ali^14)^	42	Male	Alcoholic	—	ERCP	—	EBS
2013	Doi^15)^	35	Male	Acute	—	ERCP	CBD stones^*^	EBS and PS
2014	Crinò^16)^	67	Female	Acute	—	ERCP	CBD stones	EBS
2015	Malli^17)^	56	Female	Stone	—	ERCP	CBD stones	EBS and EUS drainage
2021	Gustavo^18)^	60	Male	Stone	—	MRCP and intraoperative cholangiography	CBD stones	PP resection and hepaticojejunostomy
	Our case	64	Male	Alcoholic	Duodenum	NA	Stones in PP	PD

*After fistula formation.

CBD, common bile duct; EBS endoscopic biliary stenting; EPS, endoscopic pancreatic stenting; EPST, endoscopic pancreatic sphincterotomy; ERCP, endoscopic retrograde cholangiopancreatography; EUS, endoscopic ultrasound; F/U, follow-up; MRCP, magnetic resonance cholangiopancreatography; NA, not applicable; PD, pancreaticoduodenectomy; PP, pancreatic pseudocyst

Based on the clinical episodes and pathological findings of the present case, a pancreatic pseudocyst and groove pancreatitis with fistula formation in the common bile duct were suspected. This case is unique because the pseudocyst was filled with microbiliary stones.

Bilirubin calcium stones in the pseudocyst indicated that bile flowed into the pancreatic pseudocyst, suggesting a fistula between the pancreatic pseudocyst and common bile duct. Fistula formation between a pancreatic pseudocyst and the common bile duct may occur through erosion caused by pressure or inflammatory spread from the pseudocyst.^10)^

A total of 6 of the 14 reported cases included common bile duct stones coexisting with a fistula between the pseudocyst and the bile duct. Doi et al. reported the rapid appearance of bile-colored calcium–fatty acid stones in the common bile duct after fistula formation.^15)^ During the onset of pancreatitis, fat necrosis can lead to the deposition of saponified substances formed by the binding of calcium and fatty acids that result from the breakdown of triglycerides and other lipids. These substances may accumulate within the pancreatic pseudocyst.^19)^ In the present case, the absence of gallbladder and common bile duct stones supported the hypothesis that bile entered the pancreatic pseudocyst and bilirubin calcium stones formed within the pseudocyst.

However, in this case, neither imaging nor the pathological examination identified a fistula between the pancreatic pseudocyst and common bile duct. The pseudocyst contained only a small amount of clear colorless fluid without evidence of bile. Additionally, the minimal fluid content of the pseudocyst was atypical. Miyanishi et al. described the case of a pancreatic pseudocyst that fistulized with the common bile duct and subsequently shrank; thereafter, the fistula closed, resulting in spontaneous resolution of the pseudocyst.^8)^ Regarding the present case, although the pancreatic pseudocyst progressively shrunk and the fistula narrowed after its formation, we hypothesized that the pancreatic pseudocyst remained because of the presence of stones.

Pancreatic pseudocysts cause bleeding into the cyst, abdominal cavity, adjacent GI tract, bile duct, or main pancreatic duct. In this case, the edematous and hemorrhagic duodenal mucosa was likely a complication of groove pancreatitis and the pseudocyst.

Groove pancreatitis is characterized by the presence of pancreatic pseudocysts in the groove area and inflammatory extension into the duodenal wall, and it is strongly associated with chronic alcohol consumption. In the present case, groove pancreatitis was considered to be induced by excessive alcohol intake and resulted in the formation of a pancreatic pseudocyst in the groove region. We believe that the pseudocyst gradually enlarged, compressed the common bile duct, and caused inflammatory spread, thus leading to fistula formation. Additionally, we believe that the fistula allowed bile to flow into the pseudocyst, resulting in the formation of bilirubin calcium stones. After fistula formation, the pseudocyst likely contracted and discharged its fluid, leaving behind the stones. The fistula may have closed spontaneously or narrowed to the point of being undetectable. This sequence of events was hypothesized based on clinical findings observed after duodenal bleeding.

A retrospective analysis of imaging studies revealed the acoustic shadow of the pseudocyst caused by the stones, the absence of Doppler signals on abdominal ultrasound and EUS images, hyperdense stones without enhancement on CT images, and a clear distinction between the hypointense stones and hyperintense fluid on T2-weighted MRI images. These findings enabled the diagnosis of this pathology. If the fistula had been present preoperatively, then ERCP may have demonstrated contrast flow from the common bile duct into the pseudocyst, potentially leading to a preoperative diagnosis. Among 14 previously reported cases, ERCP identified a fistulous tract in 13 cases and MRCP combined with intraoperative cholangiography identified a fistulous tract in one case. As clinicians, we interpret test results based on our knowledge and clinical experience and consider differential diagnoses. However, it is extremely difficult to infer a disease or pathological condition that is unanticipated or has not been previously reported based on diagnostic findings alone. By contrast, if we had known that pancreatic pseudocysts can become filled with stones, then a preoperative diagnosis of the present case would have been straightforward. Therefore, we consider this case significant. Additionally, the IPMN was not retrospectively identified using MRCP or EUS. A grossly visible IPMN was not observed in the resected specimen; however, it was incidentally found during the microscopic examination. Mapping (**Fig. 6**) of the anatomical relationship of the duodenum, IPMN, and pseudocyst led us to believe that the IPMN tissue was incidentally obtained via the puncture route during EUS tissue acquisition that targeted the pseudocyst.

The clinical presentation of the present case did not align with that of a typical pancreatic tumor; furthermore, this condition was not considered as the differential diagnosis of a groove area mass. Consequently, an atypical pancreatic groove tumor and groove pancreatitis were preoperatively diagnosed, and SSPPD was performed. Recent reports indicated that 9 of 14 cases of pancreatic pseudocysts with fistula formation involving the common bile duct were successfully treated with endoscopic stent placement alone. However, the present case involved a groove area pseudocyst filled with biliary stones and duodenal bleeding. Endoscopic drainage alone would have been insufficient for removing the biliary stones from the cyst or controlling the bleeding; therefore, surgical intervention was the appropriate treatment choice. If a preoperative diagnosis had been established, and if an IPMN had been absent, then we would have chosen cyst enterostomy. Surgical stone removal and a cyst–GI anastomosis for drainage may be valid therapeutic options. However, in such cases with an IPMN, cyst enterostomy is associated with the risk of leaving behind a potentially malignant lesion. If the IPMN subsequently progresses to malignancy, then SSPPD is required. In such cases, the surgical risks may increase because of anatomical alterations and adhesions resulting from the prior cyst enterostomy and fibrosis caused by chronic pancreatitis and tumor progression. Therefore, we believe that sufficient explanation should be provided to the patient, and SSPPD should be performed at the time of diagnosis. Additionally, because the IPMN was distant from the pancreatic pseudocyst and not associated with cyst formation, its relationship with the current condition was considered negligible (**Fig. 6**).

## CONCLUSIONS

Pancreatic pseudocysts can form fistulas that involve the common bile duct, resulting in pseudocysts filled with stones that mimic pancreatic groove tumors. Accurate recognition of this rare pathology is crucial for its appropriate diagnosis and management.
